# Dataset for classifying and estimating the position, orientation, and dimensions of a list of primitive objects

**DOI:** 10.1186/s13104-022-06155-4

**Published:** 2022-07-28

**Authors:** Alireza Makki, Alireza Hadi, Bahram Tarvirdizadeh, Mehdi Teimouri

**Affiliations:** 1 Department of Mechatronics Engineering, Faculty of New Sciences and Technologies, University of Tehran, P.O. Box: 1439957131, Tehran, Iran; 2 Department of Network Science and Technology, Faculty of New Sciences and Technologies, University of Tehran, Tehran, Iran

**Keywords:** Object detection dataset, Object dimensions estimation, Orientation detection, Pose detection, Primitive object, Sim-to-real transfer

## Abstract

**Objectives:**

Robotic systems are moving toward more interaction with the environment, which requires improving environmental perception methods. The concept of primitive objects simplified the perception of the environment and is frequently used in various fields of robotics, significantly in the grasping challenge. After reviewing the related resources and datasets, we could not find a suitable dataset for our purpose, so we decided to create a dataset to train deep neural networks to classify a primitive object and estimate its position, orientation, and dimensions described in this report.

**Data description:**

This dataset contains 8000 virtual data for four primitive objects, including sphere, cylinder, cube, and rectangular sheet with dimensions between 10 to 150 mm, and 200 real data of these four types of objects. Real data are provided by Intel Realsense SR300 3D camera, and virtual data are generated using the Gazebo simulator. Raw data are generated in.pcd format in both virtual and real types. Data labels include values of the object type and its position, orientation, and dimensions.

## Objective

The concept of primitive objects means equating an object with a list of simple geometric volumes reproducible in different objects. This reproducibility helps training robots to interact with objects based on a limited list of geometric volumes in performing various robotic tasks such as grasping [1].

Our work has studied previous research in both classical and modern categories. Modern methods include research based on deep neural networks. In classical research in this field, ICP and RANSAC are among the most well-known tools, and novel research [2] has been seen frequently to improve their performance. Some problems, such as longer processing time and less accuracy than classical methods in this area, have delayed developing this group of tools with the help of deep neural networks. On the other hand, the rapid development of deep neural network tools has left researchers eager to use this method in this field [3].

After reviewing the available related resources and datasets [4–7], we could not find a suitable dataset for our purpose, mainly because of the deficiency of primitive object dimensions definition in datasets labels. On the other hand, we needed a tool that could expand the dataset at a low cost and continuously by adding new geometric objects to complete the list of primitive objects in the dataset. So we decided to create a dataset based on a simulator to train deep neural networks to classify a primitive object and estimate its position, orientation, and dimensions. Also, with novel sim-to-real transfer techniques, the accuracy of tools developed with virtual data in practical applications could be improved [3]. We have also collected real data besides virtual data with the help of a laboratory tool to evaluate the final application.

## Data description

The structure of labels in this dataset consists of 11 components as follows:

1: numbers–4, which represent the four types of primitive objects respectively for sphere, cylinder, cube, and sheet;

2–4: three components represent the object’s position with the values of x, y, and z, respectively;

5–8: four components of quaternion vector with the values of qx, qy, qz, and qw, respectively;

9–11: the three components express the dimensions of the object in meters.

The camera used in this research is an Intel-Realsense SR300 [8], which captures.pcd format images with 640 $\times$ 480 resolution. The position of the camera coordinate system in the robot base coordinate system is as follows: x = 0, y = − 0.1, z = 0.57, qx = 0.259 qy = − 0.966, qz = 0.0, qw = 0.0. The robot base coordinate system is the reference for data labeling. Virtual images are also created with the help of a plug-in virtual camera in the Gazebo simulator with 620 $\times$ 400 resolution in.pcd format.

Virtual data contains 8000 data in.pcd format, which the share of each primitive object is 2000 images. Objects with random dimensions are placed in a random position and orientation on the robot desktop in front of the camera to provide virtual data. All dimensions of primitive objects vary between 10 and 150 mm; exceptionally, the thickness of the rectangular sheets varies between 1 and 5 mm. Objects are placed on the robot desktop in front of the camera in the robot coordinate system in the width of − 300 to + 300 mm and the length of + 150 to + 300 mm. The table on which the objects are placed is 32 mm higher than the reference coordinate system. The objects in this dataset are red, the robot desktop is white, and the ground in the Gazebo simulator is gray. The light source position is constant, and the shadow mode is active in the simulator. Real data, including 200 data in.pcd format, are stored in two batches of 100 data.

This raw data in.pcd format can be used in various forms. For example, this data can be used in RGB, RGB-D, Voxel, and Unordered formats in the training process [9]. For example, the present team of researchers used simple threshold filters on color channels to segment the object in the image and stored it in Voxel format data to prepare it for deep neural network training to learn all data labels. Their suggestion was to use voxel data with Cartesian channels instead of the binary voxel in the training of this network. Another suggestion made in this study was to use distortion and noise filters on the voxel data to make the network robust to the deviation of natural objects from primitive objects, image distortions, and image noises. This dataset has been used in training and evaluating a deep neural network called POPDNet, according to Diagram 1 in the repository. This research can help readers understand this dataset’s performance clearly [10].

## Limitations

In this research, three components are used to express the dimensions of each object, but it is clear that only one component is sufficient to express the dimensions of some objects, such as a sphere. In these cases, the dimension values are repeated in all three components. For a cylinder, the first two components are duplicates and represent the diameter of the cylinder, and the third component represents the height of the cylinder.

It should also be noted that objects are placed on the robot's desktop with the help of gravity; therefore, some of the spatial orientations of objects in this dataset are lost.

The primary data in this dataset consists of the first 12 rows of Table 1, which reports images in.pcd format and labels in.txt format. Other rows in the table include voxel data and RGB data based on primary data, which have been prepared to expedite the research processes of interested researchers in.mat and.png formats. png data are prepared in size of 224 $\times$ 224 just by resizing the primary images.

**Table 1:** Overview of data files/data sets

Label	Name of data file/data set	File types (file extension)	Data repository and identifier (DOI or accession number)
Data file 1	Sphere	Archive file format (.zip) containing multiple (.pcd) files	OSF(https://doi.org/10.17605/OSF.IO/4AW5U) [11]
Data file 2	Cylinder	Archive file format (.zip) containing multiple (.pcd) files	OSF(https://doi.org/10.17605/OSF.IO/4AW5U) [11]
Data file 3	Cube	Archive file format (.zip) containing multiple (.pcd) files	OSF(https://doi.org/10.17605/OSF.IO/4AW5U) [11]
Data file 4	Sheet	Archive file format (.zip) containing multiple (.pcd) files	OSF(https://doi.org/10.17605/OSF.IO/4AW5U) [11]
Data file 5	Practical_1	Archive file format (.zip) containing multiple (.pcd) files	OSF(https://doi.org/10.17605/OSF.IO/4AW5U) [11]
Data file 6	Practical_2	Archive file format (.zip) containing multiple (.pcd) files	OSF(https://doi.org/10.17605/OSF.IO/4AW5U) [11]
Data file 7	Sphere_labels	Archive file format (.zip) containing multiple (.txt) files	OSF(https://doi.org/10.17605/OSF.IO/4AW5U) [11]
Data file 8	Cylinder_labels	Archive file format (.zip) containing multiple (.txt) files	OSF(https://doi.org/10.17605/OSF.IO/4AW5U) [11]
Data file 9	Cube_labels	Archive file format (.zip) containing multiple (.txt) files	OSF(https://doi.org/10.17605/OSF.IO/4AW5U) [11]
Data file 10	Sheet_labels	Archive file format (.zip) containing multiple (.txt) files	OSF(https://doi.org/10.17605/OSF.IO/4AW5U) [11]
Data file 11	Practical_1_labels	Archive file format (.zip) containing multiple (.txt) files	OSF(https://doi.org/10.17605/OSF.IO/4AW5U) [11]
Data file 12	Practical_2_labels	Archive file format (.zip) containing multiple (.txt) files	OSF(https://doi.org/10.17605/OSF.IO/4AW5U) [11]
Data file 13	POPDNet_diagram	Image file format (.png)	OSF(https://doi.org/10.17605/OSF.IO/4AW5U) [11]
Data set 1	Classification	Archive file format (.zip) containing multiple (.mat &.png) files	OSF(https://doi.org/10.17605/OSF.IO/4AW5U) [11]
Data set 2	Sphere_training	Archive file format (.zip) containing multiple (.mat) files	OSF(https://doi.org/10.17605/OSF.IO/4AW5U) [11]
Data set 3	Cylinder_training	Archive file format (.zip) containing multiple (.mat) files	OSF(https://doi.org/10.17605/OSF.IO/4AW5U) [11]
Data set 4	Cube_training	Archive file format (.zip) containing multiple (.mat) files	OSF(https://doi.org/10.17605/OSF.IO/4AW5U) [11]
Data set 5	Sheet_training	Archive file format (.zip) containing multiple (.mat) files	OSF(https://doi.org/10.17605/OSF.IO/4AW5U) [11]

## Data Availability

The data described in this Data note can be freely and openly accessed on Open Science Framework at https://doi.org/10.17605/OSF.IO/4AW5U. Please see Table 1 and reference [11] for details and links to the data.

## Abbreviations

ICP: Iterative closest point
PCD: Point cloud data
RANSAC: Random sample consensus
